# Prevention of Vomiting

**Published:** 1890-03-08

**Authors:** 


### THERAPEUTICS.

### PREVENTION OF VOMITING.

Andeer strongly recommends chemically pure resorcin in doses of 0·5 to 3·0 grams per diem in the treatment of vomiting of any kind and from the most diverse causes. He states that he has found it equally effective in that which attends intense pain as during the passage of biliary and renal calculi, or dysmenorrhea; in the otherwise irrepressible vomiting of seasickness and pregnancy, and even in that which follows excessive indulgence in alcohol or food.

The last-mentioned results seem unlikely and, in many circumstances, even undesirable, so long as the vomiting is a consequence of gastric catarrh or gastritis, and not a remote effect of poisoning of the nerve centres. But for all vomitings having their origin in conditions arising outside of the alimentary canal, we believe that the most effective sedatives will be found within this chemical group. For more than twenty years we have found the sickness of pregnancy yield almost invariably to gr. ½ gr. i. doses of pure phenol in water or chloroform water ℥ i.; and it has proved scarcely less successful in that following morphia injections, aural vertigo, and other forms. Originally we used creosote, but pure phenol is greatly to be preferred, if only on the ground of its perfect solubility.

